# Cadherin Signaling in Cancer: Its Functions and Role as a Therapeutic Target

**DOI:** 10.3389/fonc.2019.00989

**Published:** 2019-10-04

**Authors:** Weina Yu, Li Yang, Ting Li, Yi Zhang

**Affiliations:** ^1^Biotherapy Center, The First Affiliated Hospital of Zhengzhou University, Zhengzhou, China; ^2^Cancer Center, The First Affiliated Hospital of Zhengzhou University, Zhengzhou, China; ^3^Henan Key Laboratory for Tumor Immunology and Biotherapy, Zhengzhou, China; ^4^School of Life Sciences, Zhengzhou University, Zhengzhou, China

**Keywords:** cadherin signaling, tumorigenesis, tumor progression, angiogenesis, tumor immune response, therapeutic target

## Abstract

Cadherin family includes lists of transmembrane glycoproteins which mediate calcium-dependent cell-cell adhesion. Cadherin-mediated adhesion regulates cell growth and differentiation throughout life. Through the establishment of the cadherin-catenin complex, cadherins provide normal cell-cell adhesion and maintain homeostatic tissue architecture. In the process of cell recognition and adhesion, cadherins act as vital participators. As results, the disruption of cadherin signaling has significant implications on tumor formation and progression. Altered cadherin expression plays a vital role in tumorigenesis, tumor progression, angiogenesis, and tumor immune response. Based on ongoing research into the role of cadherin signaling in malignant tumors, cadherins are now being considered as potential targets for cancer therapies. This review will demonstrate the mechanisms of cadherin involvement in tumor progression, and consider the clinical significance of cadherins as therapeutic targets.

## Introduction

Cadherin was first described by Hyafil and Peyrieras in the 1980s as “Uvomorulin” in mouse and L-CAM in chicken (1, 2) and the mouse gene was later cloned (3). Subsequently, several other cadherins were found and analyzed, including N-cadherin (CDH1) (4), which is mainly expressed in neural tissue, and P-cadherin (CDH3), which was first characterized from placental tissue (5). Many subtypes have since been identified in other species and there are now more than 100 known members in this superfamily (6).

Functionally, cadherins act as modulators during organism growth. First, tissue cohesion in organisms depends largely on cadherins. Radice et al. (7) found that mice lacking N-cadherin (CDH2) developed heart defects and died *in utero*. Moreover, cadherins affect cell polarization, by differentiating between cell populations during development (8); this function might has a relationship with intrinsic characteristics of the ectodomains of cadherins (9).

The research to date has focused on the relationship between cadherins and malignant tumors, and drugs targeting cadherins have been developed and tested in clinical trials. In this review, we intend to summarize the mechanisms whereby cadherins mediate tumor formation and progression, and to discuss their potential use in clinical treatment of cancer patients.

## Types of Cadherin

**Figure 1:**
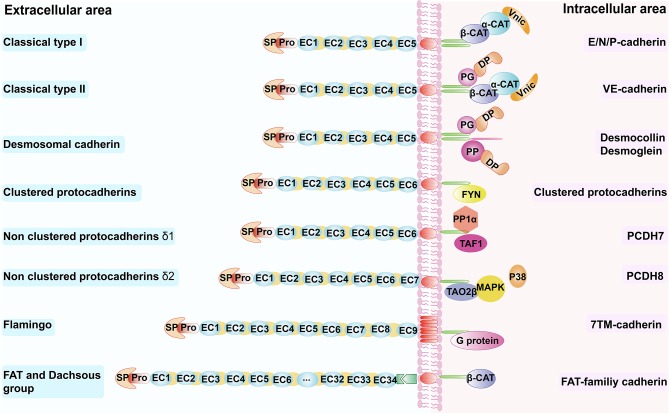
Schematic overview of the cadherin superfamily. Schematic overview of the cadherin superfamily depicting representative molecules for the respective subfamilies. β-CAT, β-catenin, α-CAT, α-catenin; Vinc, vinculin; DP, desmoplakin; PG, plakoglobin; PP, plakophilins; TAF1, template-activating factor 1; TAO2β, thousand and one amino acid protein kinase 2β; EC, extracellular cadherin repeats; SP, signal peptide; Pro, pro-peptide; FYN, Src-family kinase; PP1α, protein phosphatase-1α; MAPK, mitogen-activated protein kinase.

Cadherins can be classified into several subtypes: type I classical cadherins such as E-cadherin, N-cadherin, and P-cadherin; type II classical cadherins such as VE-cadherin (CDH5) and OB-cadherin (CDH11) (9, 10); the desmosomal cadherins (11, 12); the seven-pass transmembrane cadherins (13, 14); FAT and dachsous (DCHS) group cadherins (15–17); and protocadherins (PCDHs) (18–21) (Table 1). Nearly all cadherins are transmembrane proteins with three components: (1) an extracellular cadherin domain (EC) responsible for homotypic cadherin-cadherin interaction; (2) a single-pass transmembrane domain (absent in seven-pass transmembrane cadherins); and (3) a cytoplasmic domain acts as a connector between cell surface and cytokeleton (21, 29) (Figure 1).

**Table 1:** The cadherin superfamily.

**Subfamily**	**Example**	**Characteristics**	**References**
Type I classical cadherins	E-cadherin	E-cadherin forms the key functional component of adherens junctions of all epithelial cell and it play a vital role in the establishment and maintenance of intercellular adhesion, cell polarity, and tissue architecture.	(8)
	N-cadherin	Maintaining the proper architecture of certain tissues (structural–adhesive function), but it also plays a role in cell communication (signaling function), being involved in the establishment of functional synapses in neurons and in the formation of a vascular wall that is essential for vascular stabilization	(8)
	P-cadherin	Exhibits a singular pattern of expression co-localizing partially with E-cadherin and being restricted to the basal proliferative cell layer of the majority of stratified epithelia	(22)
Type II classical cadherins	VE-cadherin	Mainly expressed in endothelial cell, where it plays a vital role in vascular integrity and permeability and promotes homotypic cell-cell adhesion	(23)
	OB-cadherin	Mediate homophilic cell-cell adhesion and it is also involved in many other biological functions, including cytoskeletal organization, tissue morphogenesis, cellular migration, and invasion	(22)
Desmosomal cadherins	Desmogleins Desmocollins	Desmosomes are adhesive intercellular junctions and linkers of the intermediated filament cytoskeleton and are abundant in stress-bearing tissues including cardiac muscle and stratified epithelia and are composed of members from the three groups of molecules, the cadherins, armadillo proteins, and plakins	(12, 24)
Seven-pass transmembrane Cadherins	Flamingo CELSR1	Plays multiple roles in controlling epithelial and neuronal cytoarchitecture and functions in ectoderm patterning, reproductive system, and cilia development and organization	(14, 25–27)
FAT and Dachsous group	Fat cadherin Dachsous	Regulation of tissue growth through the Hippo pathway and directional control of cellular morphology and behavior, called planar cell polarity (PCP)	(15, 17)
Protocadherins	C-Pcdhs NC-Pcdhs	Protein structure and properties, gene regulation, and *in vivo* functions of the clustered Pcdh family indicate that these diverse proteins play significant roles in the vertebrate brain	(18, 28)

Classical cadherins like E-cadherin, P-cadherin and N-cadherin, which translate the intercellular contact signals involved in cellular organization, are mediated mainly by the catenins, namely α-catenin, β-catenin, and γ-catenin, which are membrane undercoat proteins that link the tail of cadherins in the cytoplasm (30, 31) (Figure 1). VE-cadherin binds to other proteins such as plakoglobin, which binds and recruits a desmosomal plaque protein named desmoplakin to the cell surface (22); vinculin also participates in this process. In tissues that undergo greater mechanical strain, such as the epidermis and myocardium, desmosomal cadherins function through plakoglobin, desmoplakin, and the armadillo family member plakophilins, to mediate the linkage between the cytoskeleton and these proteins (21). Protocadherin can be divided into two subfamilies—clustered and non-clustered protocadherins (Table 1). Clustered protocadherins have a unique binding site for non-receptor tyrosine kinase Fyn, one of the Src kinases families in the cytoplasm. Non-clustered protocadherins include three types: δ1 (e.g., PCDH7), δ2 (e.g., PCDH8), and ε (e.g., PCDH15). PCDH7 has three motifs, all of which can interact with the histone-regulating protein template-activating factor 1 and protein phosphatase-1α, and is inactivated when it binds to the CM3 motif. PCDH8 can interact with thousand and one amino acid protein kinase 2β through its intracellular domain, thereby activating the p38 MAPK pathway, which may enhance endocytosis of N-cadherin (32) (Figure 1). The flamingo cadherin differs from the other cadherins—it has a transmembrane domain that spans the plasma membrane seven times, somewhat like that of G proteins, and participates in many cellular signaling pathways (33) (Figure 1). Fat and dachsous cadherins are very large cell-adhesion molecules (Table 1); a recent study showed the involvement of Fat cadherin in the Wnt-signaling pathway with the help of β-catenin (34) (Figure 1).

## Cadherins and Tumorigenesis

In the developmental process of multicellular organisms, the maintaining of cellular and tissue morphogenesis mainly depends on cell-cell adhesion (35), which initiated and maintained by adheren junction. The signaling mediated by adhesion between cell-cell and cell-extracellular matrix is associated with gene regulation in normal tissue homeostasis (36, 37). Disturbance of cell-cell and cell-extracellular matrix adhesion and the subsequent changes in adhesion-mediated signaling pathways would induce malignant phenotypes in normal cells (38).

As a major contributor to cell–cell adhesion in epithelial tissues, abnormal expression of cadherins is closely related to tumorigenesis. β- and γ-catenin (39, 40) are bridges that mediate linkage of classical cadherins to the actin cytoskeleton through their cytoplasmic domains (41). It has been shown that cell–cell adhesion is markedly reduced when a well-differentiated benign adenoma with apico-basal polarization, a feature of normal epithelial cells, becomes an invasive carcinoma by losing its normal membrane polarization (42, 43). Many signaling pathways are involved in this process (44). Epithelial–mesenchymal transition (EMT), a phenotype transition, is a driving force in tumorigenesis (43, 45, 46).

E-cadherin and N-cadherin are founding members of the cadherin superfamily and act as crucial regulators in the process of tumor development. E-cadherin is essential in maintaining epithelial tissue integrity and providing strength to maintain polarization of the epithelial cell layers (6). N-cadherin is highly expressed in mesenchymal cells and neural tissue (7). N-cadherin promotes increased cell motility and migration, by interacting with epidermal growth factor receptor (EGFR) 1 via the 88-amino acid region of the EC4 domain (47). During malignancy, cell–cell adhesion mediated by E-cadherin is lost (48), and this process can be reversed when E-cadherin is re-established (48, 49). These studies propose the novel view that EMT can participate in the development of malignant tumors.

**Figure 2:**
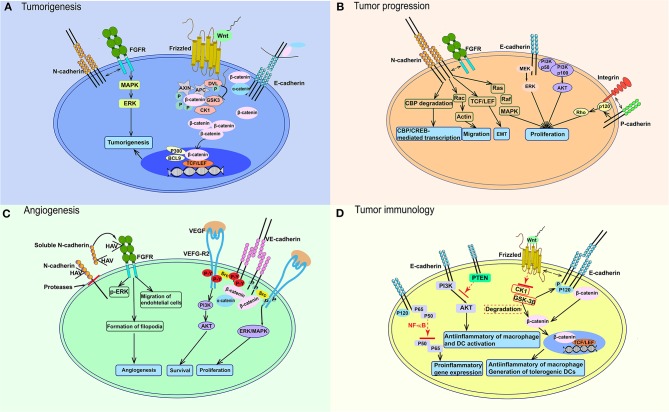
Signaling pathways participating in the function of cadherins in cancer. **(A)** E-cadherin loss lead to the upregulation of β-catenin in cytoplasm. After Wnt binds to Frizzled, it blocks the effects of CK1α and GSK3 on β-catenin and cause the accumulation of β-catenin in cytoplasm and nucleus, which activates TCF/LEF and co-activators. N-cadherin could affect tumorigenesis through interacting with FGFR and stimulating MAPK/ERK pathway. **(B)** Wnt-β-catenin pathway also works in tumor progression like in **(A)**. E-cadherin could also activate PI3K-AKT and MEK-ERK pathways. N-cadherin activates Ras-MAPK pathway, TCF/LEF transcription factor, etc. P-cadherin could increase tumor migration through interacting with integrin. **(C)** In endothelial cells, VEGF-VEGFR2 activates VE-cadherin and Src, then leads to VE-cadherin phosphorylation on tyrosines, which would promote endothelial cell proliferation through PI3K/AKT signaling. ERK/MAPK signaling pathway would be stimulated without tyrosines phosphorylation. sN-cadherin induces by proteases could bind to FGFR and phosphorylates ERK to stimulate angiogenesis. **(D)** The Wnt-β-catenin signaling pathway also works in immune cells, which stimulates anti-inflammatory macrophages and tolerogenic DCs. E-cadherin mediates anti-inflammatory activation of macrophages and DCs through PI3K/AKT and NF-κB. FGFR, fibroblast growth factor receptor; DVL, disheveled protein; P, phosphorylation; APC, adenomatosis polyposis coli; MAPK, mitogen-activated protein-kinase; ERK, extracellular signal-regulated kinase; GSK3, glycogen synthase kinase; BCL9, B-cell lymphoma 9; TCF/LEF, T-cell factor/lymphoid enhancer factor; PI3K, phosphatidylinositol 3-kinase; AKT, protein kinase B; CBP, CREB-binding protein; HAV, His–Ala–Val; VEGF, vascular endothelial growth factor; VEGF-R2, vascular endothelial growth factor receptor 2; P-Y, phosphorylation on tyrosin; PTEN, gene of phosphate and tension homology deleted on chromsome ten; NF-κB, nuclear factor κB; CK1α, casein kinase 1 α; TGF-β, Transforming growth factor beta.

Many factors can lead to E-cadherin loss. Some of cancer associated germline mutations had been reported to interfere the expression and function of cadherin (50–52). Additionally, in many epithelial malignancies like esophagus, melanoma, and hepatocellular carcinoma, the expression of E-cadherin is decreased by the hypermethylation of promoter (53). Besides, the frequent loss of p120 expression would cause the degradation of E-cadherin in lung cancer (54, 55). As a result, the expression of β-catenin would upregulate along with the reduction of E-cadherin and downstream signaling pathways associated with tumorigenesis in nuclear would be activated by the elevated β-catenin. However, E-cadherin loss by itself is insufficient to stimulate the following signaling pathway due to the decrease mechanism of β-catenin which mediated by Wnt signaling pathway (Figure 2A). In this degradation process, glycogen synthase kinase (GSK) phosphorylates β-catenin and induces its ubiquitination and degradation in the 26 S proteasome to keep the low level of β-catenin in cytoplasm, as well as cell nucleus (56). After Wnt proteins bind to Frizzled, the signaling pathway is activated (Figure 2A). The disheveled protein then binds, resulting in failure of degradation of the complex and block the degradation effects casein kinase 1 α and GSK3 on β-catenin. Thus, β-catenin accumulates in the cytoplasm and is translocated to the nucleus, where it competitively inhibits a suppresser of T-cell factor/lymphoid enhancer factor (TCF/LEF) and could active Wnt-responsive genes. Additionally, some co-activators in transcription, such as p300, CREB-binding protein and B-cell lymphoma 9, could be stimulated at the same time. N-cadherin has a physical association with and could stimulate the MAPK/ERK signaling pathway, which participates in tumorigenesis (Figure 2A).

One possible explanation for the role of EMT in this transition is that ectopically expressed N-cadherin may promote the migration of epithelial cells with a similar cadherin expression profile, and thereafter act in an entirely different way (57). On the other hand, altered cadherin expression may affect intracellular signaling, causing depolarization of cells and enhanced migratory properties (58). Many factors participate in this process. For example, The E-cadherin/β-catenin complex functions as an intercellular junction and is regulated by phosphorylation. Phosphorylation by Src or epithelial growth factor receptor (59) destroys the binding between β-catenin and cadherin, and subsequently enhances the expression of cytoplasmic β-catenin, which activates Wnt/β-catenin signaling. It has been reported that abnormal activation of this pathway may induce tumor formation, by binding with TCF/LEF-type transcription factors and stimulating cancer-related genes such as *c-MYC* and *CCND1*, which may affect the cell cycle and promote tumorigenesis (Figure 2) (60). N-cadherin has also been reported to be physically associated with various members of the fibroblast growth factor receptor (FGFR) family, and to participate in increasing the cell-surface receptor level and sustaining MAPK/ERK signaling, thus increasing cellular motility and invasion (61, 62).

Other types of cadherins have similar effects in malignant tumor formation. For example, the role of P-cadherin in human cancer has been debated for years (63, 64). Moreover, loss of P-cadherin has a significant correlation with histological tumor staging (65). In a P-cadherin-null mouse model, mammary glands developed hyperplasia, dysplasia, and abnormal lymphoid infiltration (66), demonstrating that P-cadherin expression is important in maintaining normal tissue. Similarly, Hardy et al. (67) found that in colonic lesions, regulation of P-cadherin differs from that of E-cadherin during colonocyte transformation; P-cadherin was expressed in low-malignancy potential lesions, indicating that P-cadherin is unlikely to participate directly in tumorigenesis. Similarly, H-cadherin participate in the carcinogenesis of colorectal cancer (68).

Therefore, cadherins function not only as the molecules of cell-cell adhesion but also as factors which induce tumorigenesis predominantly through Wnt/β-catenin or other signaling pathways.

## Cadherins and Tumor Progression

Once cell-cell adhesion was disrupted, adhesion-mediated signaling pathways would be changed subsequently. Some characteristics in invasive metastatic cancer, such as cell mobility, would be affected as well (35). Cadherins have been implicated in tumorigenesis, and research into their roles in tumor progression should not be neglected. It is widely accepted that EMT of epithelial cells results in the feature of strong cell-cell adhesion and more motile and invasive characteristics (69, 70). A feature of EMT is the downregulation of E-cadherin and parallel upregulation of other cadherins, like N-cadherin, which plays a key role during early invasion and metastasis (71, 72).

### E-cadherin and Tumor Progression

Many researches have proved that E-cadherin expression is often lost in advanced tumors; this may be associated, at least in some instances, with increased metastasis and recurrence (73). It had been demonstrated *in vitro* that downregulation of E-cadherin is related to the loss of epithelial phenotype (74, 75) and the acquisition of the mesenchymal phenotype, which is associated with invasive behavior; this has been validated using 3D gels and heart explants (76). Further, partial or complete reversal of this process occurs when E-cadherin is constitutively produced (49, 74, 77–79).

The E-cadherin–catenin complex was the first signaling pathway to be studied. Many factors affecting this complex participate in reduced cell adhesion, which is of great importance during tumor metastasis (80); these factors include reduction, loss, or redistribution of E-cadherin, and mutation or reduced transcription of the related genes (81). Similarly, many factors lead to downregulation of E-cadherin-mediated cell adhesion; these include hepatocyte growth factor, epithelial growth factor, and TGF-β, which are thought to initiate EMT and upregulate EMT-related transcriptional factors such as Snail, Slug, and T-wist (82–86) (Figure 2). This downregulation reduces cell–cell adhesion, and an oncogenic stimulus via β-catenin, further stimulating the canonical Wnt signaling pathway, and finally stimulating TCF-regulated genes. TCF-regulated genes, which are key factors in cell proliferation and invasion, including *CCND1, MMP-7, MMP14, FN1, c-MYC, LGR5, PLAUR* etc. (87–90). The non-canonical Wnt pathway may also influence cancer metastasis, and overexpression of Wnt5a is responsible for enhanced cell migration and resistance to treatment (91, 92) (Figure 2). Further, crosstalk involved in cell–cell communication and cell-matrix adhesion can be regulated by several signaling pathways that affect the migratory and invasive ability of cells by disrupting adherens junctions when integrins are activated (93). For example, a downstream factor of integrin focal adhesion kinase–Src regulates TGF-β-induced EMT (94), and is involved in coupling integrin activation with TGF-β-induced EMT (95–97). E-cadherin also plays a role in cancer progression itself, partially through the PI3K-AKT and MEK-ERK pathways. Integrin functions as a cell–cell adhesion molecular and its interaction with P-cadherin increases migration (Figure 2B).

However, Kowalski et al. (98) hold the view that abnormal E-cadherin expression is more common in invasive ductal carcinomas with the potential to develop distant metastases. E-cadherin expression in more consistent and often more frequent in distant metastases than in the primary cancer. Therefore, it is possible that E-cadherin protein is re-expressed once cancer cells reach distant sites. In the study from Bukholm et al. (99), 95% metastatic lymph nodes were highly expressed E-cadherin. The expression or re-expression of E-cadherin in distant metastases of invasive cancer suggests that it may contribute to the establishment of metastatic foci (100).

### N-cadherin and Tumor Progression

The other key factor involved in EMT is N-cadherin. A major consequence of EMT is the loss of stable adhesive junctions of epithelial cells, the reversion of normal polarity among cells, and the destruction of epithelial tissue structure, therefore accelerating the migration of malignant tumor cells toward adjacent tissues (101, 102). Unlike E-cadherin, N-cadherin upregulation enhances the migratory and invasive capacities of tumor cells (103). N-cadherin is as important as E-cadherin in development and adulthood. Besides, during tumor progression, N-cadherin can affect cell survival, facilitate the process of EMT and migration/invasion by recruiting signaling molecules. Crosstalk of N-cadherin with other membrane protein such as fibroblast growth factor receptor (FGFR) also activates signaling cascades that affect cell proliferation, invasion, and cell–cell adhesion (Figure 2B). In normal epithelial cells, N-cadherin is absent or expressed at low levels, and aberrant expression is associated with malignancies and tumor progression (71, 104–106). Using an orthotopic mouse model, Shintani et al. (107) showed that, compared to N-cadherin cancer cell lines, N-cadherin-knockdown cancer cells formed significantly smaller primary tumors and micrometastases in lungs. A similar result was found by Bouvet et al. (108). In E-cadherin–positive cancer cell lines, overexpression of N-cadherin made cells more invasive both *in vitro* and *in vivo*, but did not cause parallel downregulation of E-cadherin (109, 110). These results suggest that N-cadherin may promote cancer metastasis even without the presence of E-cadherin (103).

Many signaling pathways participate in the process of malignancy. Hulit et al. (111) found that mammary epithelium tumors had greater metastatic potential in PyVmT transgenic mice with N-cadherin expression than in those without it. Further, PyVmT transgenic mice with N-cadherin expression showed greater ERK activation, and migration and invasion, and increased matrix metalloprotein-9 expression, than those without N-cadherin expression (111). Further, when FGFR was blocked in the PyVmT transgenic mice with N-cadherin expression, motility and invasion were reduced, as was ERK phosphorylation. Similar results have been found in several other cell lines that co-express N-cadherin and FGFR-1 (112), suggesting that the FGFR-N-cadherin signaling pathways might be general routes involved in metastasis. These results reveal a signaling cascade, N-cadherin/FGFR/MAPK/ERK/ matrix metalloprotein-9, in which N-cadherin mediates metastasis (103).

### Other Cadherins and Tumor Progression

Aside from the two classical cadherins, other types of cadherins can also participate in metastasis. Despite its role in the maintenance of tissue architecture (113, 114), studies are increasingly revealing that P-cadherin dysfunction is strongly associated with tumorigenesis and confers the malignant phenotype in cancer cells (115–118). Mandeville et al. (119) revealed that P-cadherin can modulate the migratory ability of bladder carcinoma cells *in vitro*. Similarly, Taniuchi et al. (120) demonstrated that, in the vast majority of pancreatic ductal adenocarcinoma cells examined, overexpression of P-cadherin altered the subcellular localization of the p120 catenin, and increased the steady-state activity of Rho GTPases. Those changes were associated with increased motility of pancreatic ductal adenocarcinoma cells. In the liver metastatic site of colon cancer, P-cadherin expression was much higher compared to primary site; P-cadherin knockdown affected wound healing, proliferation, and colony formation, and inhibited liver metastatic foci formation *in vivo* (121).

OB-cadherin belongs to the type II cadherin subfamily, and is related to a poor prognosis (104, 122). OB-cadherin interacts with α- and β-catenin *in vivo* (122, 123) and may play a role in connecting to the cell-matrix, especially in promoting cell extension. Therefore, OB-cadherin may participate in promoting invasion and metastasis of malignant tumor via the interaction with fibroblasts or osteoblasts in the stroma and bone (122).

Bartolomé et al. pointed out that the Arg-Gly-Asp motifs of VE-cadherin activate the α2β1 integrin, and then promote cell adhesion, cancer invasion, proliferation, and lung metastasis (124). It has been revealed that higher expression of VE-cadherin leads to poor prognosis in breast cancer and melanoma patients. These results suggest the novel concept that therapies that target cadherin-specific Arg-Gly-Asp motifs may control distant metastasis in multiple cancers.

The role of PCDHs in cancer progression is complicated. Several studies have mentioned that PCDHs could induce apoptosis of tumor cells and prevent malignant tumor from proliferating and metastasis (125). This point of view has been approved in colon cancer (126). Similarly, based on *in vitro* testing, restoration of PCDH9 expression has effects on cell cycle and could strength apoptosis (127). So do PCDH20 and PCDH17 (128–130). When PCDHs are lost, tumor cells gain a more aggressive phenotype. For example, loss of PCDH10 significantly enhances the proliferation of myeloma cells (131) and may act as a prognostic marker for the reduced survival of colorectal cancer patients (132). More specifically, when PCDH17 was lost, it could stimulate EGFR/MEK/ERK signaling pathway and accelerate the progression of liver cancer (130). Recent research has elucidated the oncogenic activity of PCDHs. It has been reported that PCDH7 is significantly increased in breast cancer and has a positive correlation with brain metastases (133). Further, Chen et al. and Terry et al. found PCDH-PC, a cytoplasmically retained variant of PCDH11Y, was upregulated in advanced prostate cancer and metastasis (134, 135). The specific mechanisms whereby PCDHa affect cancer remain to be studied.

In brief, cadherins, especially E-cadherin and N-cadherin, participate in EMT and thus regulate tumor invasion and metastasis.

## Cadherins and Angiogenesis

Malignant tumors consume much more energy and oxygen than normal tissues and need fast-growing vascular networks to support the proliferation of tumor (136). In this process, various types of pro-angiogenic factors are secreted by tumor cells and stromal cells and help building abnormal vascular networks which are disorganized, immature and permeable, leading to inadequately perfused malignant tumors (137). In the development of malignant tumor, cadherins do more than just providing adhesion. They interact with many cytoskeletal and signaling molecules by transferring signals into cells. Endothelial cells present different cadherins which may transfer specific signals and have distinct functional roles.

VE-cadherin, originally called cadherin-5 (138), is mainly located in vascular endothelial cells and is indispensable for vascular building during embryonic development (139, 140). In addition to being a transmembrane component of the endothelial cadheren junction, it maintains the integrity and permeability of the endothelium and vascular system (141, 142). Thus, a burst of VE-cadherin expression may be required in promoting and sustaining vascular growth.

VE-cadherin promotes tumor progression mainly by facilitating tumor angiogenesis (143). VE-cadherin could interact with vascular endothelial growth factor receptor (VEGFR)-2 (Figure 2C) and stimulate TGF-β signaling pathway to enhance cell proliferation (144). Many growth factors such as VEGF could induce the tyrosine phosphorylation of VE-cadherin, β-catenin and p120, which would increase vascular permeability *in vitro* system (145). VE-cadherin phosphorylation through the VEGF/VEGF R2 pathway needs Src activity (146) (Figure 2C). In angiogenic tissues, VE-cadherin-associated Src phosphorylation is increased (143) and inhibition of Src inhibits VEGF-stimulated vascular permeability (147). With the help of other phosphatases, VEGF 2R and Src lead to the phosphorylation of VE-cadherin on tyrosines (143) and this association would induce endothelial cell survival through PI3K/AKT pathway (Figure 2C). However, in the absence of protein tyrosine phosphatase activity, ERK/MAPK pathway is activated to stimulate cell proliferation (Figure 2C) (148). Similarly, VE-cadherin can interact with the TGF-receptor complex (149), platelet-derived growth factor β-receptor (150), or FGFR1 (151), among others, and may affect the proliferation of endothelial cells and stability of vascular structures.

N-cadherin functions mainly as a promoter of malignant tumors. However, the role it plays in tumor angiogenesis should not be underestimated. N-cadherin affects vascular tissue mainly by interacting with pericytes and vascular smooth muscle cells in the endothelium (152, 153). Unlike VE-cadherin, which predominates in vascular epithelial cells, N-cadherin predominates in pericytes. In chickens, once N-cadherin is neutralized or lost, defective pericyte adhesion occurs causing aberrant vascular development (154). The amount of VE-cadherin expressed depends on N-cadherin, and N-cadherin affects vascular morphogenesis by regulating VE-cadherin (155), suggesting that N-cadherin is a crucial regulator of angiogenesis.

Nalla et al. (156) found that N-cadherin knockdown suppressed the expression of several angiogenic molecules and reduced the angiogenic ability of PC3 cells (156), which indicates that endothelial cell is not the only target of N-cadherin, angiogenesis could also be promoted by N-cadherin through regulating chemokines and angiogenic molecules. Doherty et al. (157, 158) have found that N-cadherin has an His–Ala–Val (HAV) sequence in the extracellular domain directly linked to the HVA sequence present on FGFR (Figure 2C) and propose that trans-interactions between N-cadherin molecules on cell membranes trigger cis-interactions between N-cadherin complexes and FGFR monomers, subsequently causing FGFR dimerization and activation. Thus, N-cadherin-mediated endothelial cell adhesion may facilitate angiogenesis. Similarly, a soluble 90 kDa fragment of N-cadherin promoted angiogenesis in an FGFR-dependent manner (159). Proteases cleave the extracellular part of N-cadherin and produce soluble N-cadherin. Soluble N-cadherin can phosphorylate ERK and stimulate angiogenesis (Figure 2C).

In conclusion, cadherins play a vital role in tumor angiogenesis mainly through binding to VEGFR or platelet-derived growth factor receptor in order to affect vascular structures.

## Cadherins and Tumor Immune Response

A tumor is not a one step result; the role of host immunity in the formation and progression of maglinant tumor s is complex and should not be negelected. Solid tumors are commonly infiltrated by a large number of immune cells which funtion seems pretty paradoxical (81). Once the microcolonies of malignant cells were detected by immune system, innate and adaptive immune cells would be able to eradicate them. However, genome plasticity renders some tumor cells the ability to proliferate continuously and thus cause an equilibrium between the generating and eradicating of tumor cells. The continuing proliferation of tumor cells would finally cause a broken equilibrium along with detectable tumor followed by metastasis (160). Concomitantly, surviving tumor clones control immune responses by specifically suppressing anti-tumor effector mechanisms and accumlating immunosuppressive immune cell subsets, inculding DCs, tumor-associated macrophages and Tregs at the tumor site (81).

Viollet et al. (161) hold the view that E-cadherin participates in the maintaining of epithelial barrier and prevents potentially harmful agents from inner tissue. This is the immunologic function of E-cadherin. Furthermore, E-cadherin might take part in the regulation of epithelial innate immune function through multiple chemokines and cytokines and modulating transepithelial transfer of immunes cells (162). Recent studies have shown that cadherins are highly expressed in several leukocytes, such as dendritic cells (DCs), macrophages and Langerhans cells (161).

DCs are important participators in adaptive immunity. The roles of cadherins in DCs have been investigated. It is widely recognized that E-cadherin is expressed in Langerhans cells, a type of immature DC (163). As DCs mature, their expression of E-cadherin is reduced to facilitate the migration of DCs to the lymphoid organs (164). Infiltration of DCs occurs in solid tumors, and E-cadherin expression in DCs may be upregulated by tumor cells; this upregulation is thought to inhibit the function of DCs in presenting tumor antigens (165). Jiang et al. (166) showed that bone marrow-derived DC (BMDC) express E-cadherin, and BMDC activation would be induced when E-cadherin-depended DC-DC interaction was disrupted. This would upregulate the expression of costimulatory molecules and CCR7, downregulate the macropinocytosis, and lead to the migration of MHC II molecules from lysosomes to cell surface. At the same time, loss of E-cadherin results in the accumulation of cytoplasmic β-catenin and stimulates the following signaling pathways like TCF. Manicassamy et al. (167) showed that β-catenin deletion in DC would accumulates Th1 and Th17 cell infiltation in intestine of mice, and decrease the number of regulatory T cells. This indicates that E-cadherin plays a significant role in DCs and may affect their functions in the tumor microenvironment.

Macrophages are involved in the whole process of life via phagocytic ability and producing immunoregulatory mediators and trophic. They could also recognize, engulf and destruct pathogens to facilitate tissue development and recognition and maintain homeostasis (168). According to the research of Rehli et al. (169), IL-4 could induce the expression of E-cadherin, which was considered as a marker of M2 macrophages, in mouse bone marrow-derived macrophages (*in vitro*) and several mice models (*in vivo*) (170).

In specimens of sarcoidosis and foreign body granulomas from humans, E-cadherin is expressed in skin granuloma macrophages (171). In an *in vitro* experiment, IL-4/IL-13 induced macrophages to express E-cadherin (172, 173). In Th2-driven diseases, IL-4 and IL-3 are crucial for inducing E-cadherin in both mouse and human macrophages (172). However, in the tumor microenvironment, E-cadherin expression is higher and independent of IL-4/IL-13 in M1-like macrophages, in contrast to the situation in M2-like macrophages (174). One possible reason is that M2-like macrophages are preindominantly accumulated in the hypoxic areas of tumor tissue, which reported to reduce E-cadherin expression (175). Further, E-cadherin may contribute to the process of fusion and engulfment in macrophages: β-catenin is barely detectable in naïve macrophages due to the absence of E-cadherin, but is found to be stable in the E-cadherin/β-catenin complex after stimulated with IL-4, which could enhance the fusion and engulfment of pathogens in macrophages in a STAT5-dependent way (172, 176). Thus, E-cadherin might act as a promoter in helping macrophages to kill tumor cells.

The classical cadherin pathways, including Wnt, PI3K, Rho-family GTPases, and NF-κB, operate in macrophages and DCs. Besides, Wnt/β-catenin has been reported to contribute to the anti-inflammatory phenotype of macrophages and tolerogenic state of DCs (177). When Wnt binds to Frizzled, the phosphorylation of β-catenin by casein kinase 1 and GSK-3β is inhibited, leading to an upregulation of nuclear β-catenin and activation of TCF/LEF-dependent gene expression, which stimulates anti-inflammatory macrophages and tolerogenic DCs (Figure 2D). And at the same time, other pathways can also influence macrophage and DC functioning. It has been shown that E-cadherin could suppress the activity of macrophages and DCs via PI3K/AKT signaling pathway. In addition, NF-κB, a vital transcription factor, could also be regulated by E-cadherin-catenin in macrophages and DCs, using p120 catenin as a docking site and working as a suppresser (Figure 2D).

On the surface of other immune cells, including CD8^+^ T, NK, and regulatory T cells, there are various ligands that can bind to the E-cadherin expressed on DCs and macrophages. For example, killer cell lectin-like receptor G1 (KLRG1) binds to the N-terminal homodimeric interface of E-cadherin EC1 (178). KLRG1 is expressed mainly on the surface of mature NK cells, and its activation inhibits NK cytotoxicity (179, 180). Furthermore, KLRG1 has been found on CD4^+^ T cells and CD8^+^ T cells (181, 182) and regulates T cell responses to suboptimal conditions. The integrin αE chain, also known as CD103, is a ligand expressed mainly on intraepithelial αβ and γδ T cells, and mediates the adhesion between E-cadherin^+^ epithelial cells and T cells *in vitro* (183) (Figure 2).

In shortly, besides the effect on tumor cells, cadherins also participate in regulating various immune cells in the tumor microenvironment. However, further study is needed to illuminate the role of cadherins in tumor-associated immune cells.

## Therapeutic Targets in Cancer

It has been reported that many signaling pathways or proteins are abnormally activated in malignant tumor cells (184–186), so molecular-targeted therapy has been developed and achieved pretty good response in cancer patients recent years (187, 188). Those molecular-targeted drugs are able to kill cancer cell selectively with decreased toxicity on normal cell, which is the significant improvement comparing with traditional anticancer drugs (189). Targeted drugs inhibit specific signaling pathways that contribute to the malignant phenotype of cancer cells and limit the progression of malignant tumors. Cadherins, vital molecules in tumorigenesis and cancer progression, could be also worked as therapeutic targets in cancer treatment.

### Inhibition of Angiogenesis

Generation of new vascular network tissue is of great importance for tumors because of their high proliferative rate. As a result, targeting tumor blood vessels is a feasible strategy in cancer therapy. Specific antibodies of VE-cadherin have effects on vascular permeability, which suggests that targeting VE-cadherin is feasible in controlling vascular barrier function (190). Further, blocking of VE-cadherin-mediated endothelial-cell adhesion affected microvessel stability (191, 192).

Liao et al. (193) were the first to reveal that BV13, a monoclonal antibody of VE-cadherin that binds to EC4, inhibits angiogenesis, tumor growth, and metastasis. However, administration of the monoclonal antibody (mAb) BV13 *in vivo* markedly increased permeability in the lungs and heart (194). To resolve this problem, another mAb, BV14, was developed; this did not significantly increase the permeability of the lungs, heart, and other organs, even after repeated high doses, and even in animals carrying well-developed tumors. BV14 and BV13 were equally effective in inhibiting angiogenesis in the mouse cornea and downregulating the growth of hemangioma and C6 glioma (Table 2). Corada et al. (202) tested five different mAbs directed at different extracellular domains of VE-cadherin, to investigate their functions and side effects. They found that mAb Cad5, which binds to the EC1 domain of VE-cadherin, was the most effective in increasing permeability. EC1 may be important in mediating cell–cell interaction, and thus the binding of an antibody to it would disrupt the integrity of vessels. Another mAb, E4G10, binds to the N-terminal sequences of VE-cadherin; unlike BV13, which binds to all vasculature, E4G10 binds only to the tumor neovasculature (191, 195). Therefore, future research should focus on finding ways to disrupt endothelial junctions in growing vessels, without affecting stabilized junctions in normal vessels (192, 195). These findings demonstrate that VE-cadherin plays a crucial role in postnatal angiogenesis, and further validate VE-cadherin as a potential target for antiangiogenic treatment.

**Table 2:** An overview of cadherin as a therapeutic target for cancer.

**Cadherin**	**Category**	**Example**	**Target**	**Function**	**Tumor**	**Clinical trial**	**References**
VE-cadherin	Monoclonal antibody	BV13	EC1	Inhibits angiogenesis, tumor growth, and metastasis	Lung cancer Epidermoid carcinoma Glioma (*in vivo*)	N/A	(193)
	Monoclonal antibody	BV14	EC4	Inhibits angiogenesis, tumor growth, and metastasis	Glioma (*in vivo*)	N/A	(194)
	Monoclonal antibody	E4G10	N terminal	Inhibits angiogenesis	Epidermis carcinoma Glioma (*in vivo*)	N/A	(195)
N-cadherin	synthetic linear peptides	H-SWTLYTPSGQSK-NH 2	Mimicking the natural HAVD sequence of N-cadherin	Block neurite outgrowth, myoblast fusion, and cell migration	Breast cancer (*in vivo*)	N/A	(196)
	Synthetic cyclic peptides	ADH-1	Mimicking the natural HAVD sequence of N-cadherin	Inhibit angiogenesis, metastasis, cell proliferation and tumor growth Cause apoptosis of multiple myeloma	Myeloma neuroblastoma pancreatic cancer (*in vitro*)	Phase Ib/II Phase II	(197, 198)
	Monoclonal antibody	GC4	EC1	Block tumor migration, invasion and metastasis	Myeloma (*in vivo*)	N/A	(199)
	Monoclonal antibody	1H7	EC1–3	Inhibit tumor growth and localized muscle invasion and distant lymph node metastasis	Prostate Cancer (*in vivo*)	N/A	(200)
	Monoclonal antibody	2A9	EC4	Inhibit tumor growth, localized muscle invasion, and distant lymph node metastasis	Squamous carcinoma breast cancer (i*n vivo*)	N/A	(77)
OB-cadherin	Monoclonal antibody	2C7	EC3	Inhibit the OB-cadherin-mediated aggregation efficiently and the metastasis to bone	Prostate cancer (i*n vivo*)	N/A	(201)

As with VE-cadherin, it has long been known that N-cadherin occurs on endothelial cells, but its role in vascular structure is only now becoming a focus of research. Inhibition of N-cadherin function destabilizes microvessels structure (154, 203). For example, direct blockage of N-cadherin impedes the adhesive complex between endothelial cells and pericytes, and thus induces hemorrhage (154). Several types of antagonists targeting N-cadherin have been discovered. Three types of antagonists are based on the cell adhesion recognition sequence HAV: synthetic linear peptides, synthetic cyclic peptides, and non-peptidyl peptidomimetics (204). ADH-1, a synthetic cyclic peptide harboring the HAV motif, has been shown to inhibit angiogenesis in various model systems (197), including the chick chorioallantoic membrane assay (205). When ADH-1 is used in a tumor-bearing mouse model, the hemorrhage that it causes is limited to tumor blood vessels, without affecting normal vessels. Therefore, it is possible that the antiangiogenic properties of ADH-1 induced the massive apoptosis we observed in the tumors that we studied (107). Another N-cadherin antagonist, H-SWTLYTPSGQSK-NH 2 (196), has recently been discovered; it may inhibit endothelial cell tube formation *in vitro*, indicating that it has antiangiogenic properties. In 2010, two monoclonal antibodies of N-cadherin were developed that inhibit the invasiveness and proliferation of N-cadherin-expressing cancer cells *in vitro* and suppress tumor growth and lymph-node metastasis *in vivo* (200).

Although VE-cadherin and N-cadherin are promising targets in tumor therapy, they have some shortcomings. Toxicity is one of the questions needed to be solved in the future, as they both widely expressed in normal tissues like vascular, heart, peripheral nerve and liver. Loss of VE-cadherin might cause hemorrhage and loss of N-cadherin can disrupt the structure of organs, leading to severe side effects. More precise targeting is high on the agenda, so do further preclinical and clinical testing to confirm the safety.

### Blockade of Metastasis

As N-cadherin involves in cancer metastasis, N-cadherin antagonists are showing promise as therapeutic drugs for inhibiting cell adhesion and modulating cell signaling, thus preventing tumor metastasis. As mentioned above, ADH-1 is a stable cyclic peptide harboring an HAV motif, which similarly inhibited N-cadherin-dependent functioning (197). It has been reported that ADH-1 can cause apoptosis in several cancer cells (107, 206, 207) and inhibit tumor cell migration at sub-cytotoxic concentrations (207–209). In a pre-clinical mouse model, ADH-1 not only inhibited primary tumor growth, but also prevented localized tumor invasion and metastasis (210). Further, ADH-1 has entered clinical testing, and there are two completed Phase I studies (211). Combination of ADH-1 and various cytotoxic agents has shown great promise: positive results in animal models, the absence of severe side effects, and promising antitumor effects in Phase I studies. There are also several monoclonal antibodies, such as GC4, directed against N-cadherin, that can block N-cadherin-dependent tumor migration and invasion *in vitro* and metastasis *in vivo* (199, 212, 213). Besides, GC-4-mediated blocking could increase the sensitivity to imatinib in CML, which means a potential therapeutic strategy in tyrosine kinase inhibitor resistance (214). 1H7 (targeting N-cadherin EC1–3) and 2A9 (targeting N-cadherin EC4) are newfound antibodies targeting N-cadherin and have been proved effectiveness in treating prostate cancer in mouse model. Both slowed down tumor growth and reduced local invasion and distant metastasis in lymph nodes (200, 215).

OB-cadherin might also be effective as a therapeutic target. Kaur et al. (216) found that OB-cadherin knockdown markedly reduced the survival ability of cancer cells *in vitro* and colony-forming abilities when there were no exogenous growth factors. Therefore, OB-cadherin is a potential target to reduce the aggressiveness and motility of cancer, and develop specific drugs to target it is an urgent priority. To explore this possibility, Lee et al. (201) generated a panel of monoclonal antibodies (mAbs) against the OB-cadherin extracellular domain; one of these, mAb 2C7, inhibited the osseous metastasis of PC3-MM 2 cells (Table 2).

Taken together, cadherins targeted therapy could effectively limit the progression and angiogenesis of cancer, and further studies should be focused on elevating the specificity and effectiveness, as well as the combination therapies.

## Conclusions

In summary, cadherins are key factors in maintaining normal tissue development. However, abnormal expression is found in tumorigenesis, development and metastasis. Therefore, a better understanding of cadherins is critical for cancer clinical applications, especially as therapeutic targets. In this review, we have discussed the various types of cadherins, their effects on tumor growth, and the clinical application of cadherins. Targeting cadherins is a promising strategy for cancer treatment and further studies and clinical experiments are needed to make it come true.

## Author Contributions

WY and LY contributed equally to preparing and writing the manuscript. WY and TL prepared the figures and tables. LY and YZ designed, reviewed, and edited the manuscript.

### Conflict of Interest

The authors declare that the research was conducted in the absence of any commercial or financial relationships that could be construed as a potential conflict of interest.
